# Scheduling and Rescheduling Operations Using Decision Support Systems: Insights From Emotional Influences on Decision-Making

**DOI:** 10.3389/fnrgo.2021.586532

**Published:** 2021-02-22

**Authors:** Mélody Mailliez, Olga Battaïa, Raphaëlle N. Roy

**Affiliations:** ^1^Institut Supérieur de l'Aéronautique et de l'Espace, ISAE-SUPAERO, Université de Toulouse, Toulouse, France; ^2^Kedge Business School, Talence, France

**Keywords:** scheduling, rescheduling, decision support systems, incidental emotions, uncertainty, decision-making, modern manufacturing environment

## Abstract

For many years, manufacturers have focused on improving their productivity. Production scheduling operations are critical for this objective. However, in modern manufacturing systems, the original schedule must be regularly updated as it takes places in a dynamic and uncertain environment. The modern manufacturing environment is therefore very stressful for the managers in charge of the production process because they have to cope with many disruptions and uncertainties. To help them in their decision-making process, several decision support systems (DSSs) have been developed. A recent and enormous challenge is the implementation of DSSs to efficiently manage the aforementioned issues. Nowadays, these DSSs are assumed to reduce the users' stress and workload because they automatically (re)schedule the production by applying algorithms. However, to the best of our knowledge, the reciprocal influence of users' mental state (i.e., cognitive and affective states) and the use of these DSSs have received limited attention in the literature. Particularly, the influence of users' unrelated emotions has received even less attention. However, these influences are of particular interest because they can account for explaining the efficiency of DSSs, especially in modulating DSS feedback processing. As a result, we assumed that investigating the reciprocal influences of DSSs and users' mental states could provide useful avenues of investigation. The intention of this article is then to provide recommendations for future research on scheduling and rescheduling operations by suggesting the investigation of users' mental state and encouraging to conduct such research within the neuroergonomic approach.

## Introduction

Establishing production schedules demands a detailed description and knowledge of the production process and requires handling a large amount of information (Rossit et al., 2019). Indeed, scheduling decisions are “a complex cognitive process that comprises a considerable number of interrelated subtasks” (Dimopoulos et al., 2012; p. 8–9; see Cegarra, 2008, for a cognitive typology of scheduling situations). In the modern manufacturing environment, the original schedule must be regularly updated because it takes place in a very dynamic and uncertain environment. In such an environment, it is not possible to create procedures for every disruption that might occur. Unexpected events inevitably happen and affect the original schedule requiring a time-pressured response (Battaïa et al., 2019). For example, taking too long to deal with unexpected events can lead to a significant decrease in the manufacturing system performance (Jepson et al., 2017). Production scheduling and rescheduling operations are crucial to maintain and increase the manufacturing productivity and effectiveness (Vossing, 2017), especially in identifying conflicts in production lines and anticipating the occurrence of unexpected events (Vieira et al., 2003; Rossit et al., 2019; see also Larco Martinelli et al., 2019 for a sequence of schedulers' action).

Decision support systems (DSSs) have been developed to support managers and more generally any kind of operators in charge of scheduling activities in their decision-making process (Manzey et al., 2012; Zikos et al., 2018; Onnasch and Hösterey, 2019). DSSs could help users in their analysis of the situation to reach an optimal and effective solution (Riveiro et al., 2014). Huge interest has been demonstrated in investigating the effect of DSSs on user performance (e.g., Ferris et al., 2010). DSSs could be useful for managers and operators in improving on-time delivery, increasing their responsivity (Herrmann, 2006) by providing information that may release users' cognitive demands (Lee and Seong, 2009; Onnasch and Hösterey, 2019). For instance, when using DSSs, individuals might understand and identify potential mistakes more easily (Lee and Seong, 2009). DSSs are also supposed to reduce the mental workload as they automatically reschedule the production planning (e.g., Onnasch and Hösterey, 2019). For example, Navarro et al. (2018) have shown that the subjective workload increased when participants performed all tasks exclusively by themselves compared to when they used fully automated tasks (see also Röttger et al., 2009).

However, DSSs do not necessarily imply an improvement in individuals' performance. It has been shown that they take more time to intervene when using DSSs because they have to recover situation awareness (Lee and Seong, 2009; van der Kleij et al., 2018). Although there is a strong interest in human factors within the scheduling and rescheduling literature (see, e.g., Sanderson, 1989; Crawford and Wiers, 2001; for reviews), there is also a need to deepen the understanding of operators' cognitive processes and performance (see also Smith and Geddes, 2003). This issue could be resolved by bridging the gap between laboratory and field studies. Hence, neuroergonomics, a recent field at the crossroads of several fields of study such as neuroscience, cognitive engineering, and human factors, proposes to examine the brain mechanisms that underlie human–technology interaction. Especially, this approach aims to investigate the cognitive and neural processes in the context of carrying out various real-world tasks under investigation, rather than under reduced isolated conditions that occur only in the laboratory (Callan and Dehais, 2019). In light of this statement, perspectives on overlooked factors are presented in the following section. We therefore highlight how the neuroergonomic approach can substantially improve the understanding of operators' mental states and performance during DSS use while reducing the gap between laboratory and field studies.

## Perspectives and Discussion

**Figure 1:**
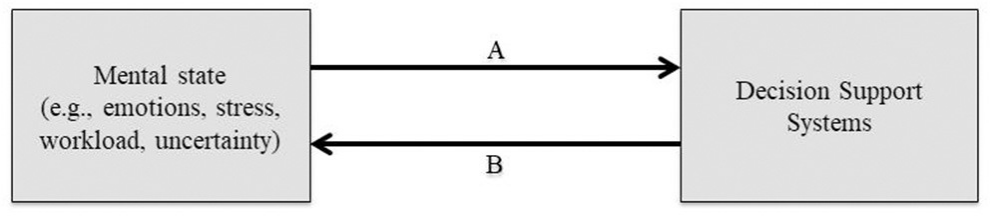
Representation of the reciprocal influence of DSSs and users' mental state during (re)scheduling production operations. Line A: influence of users' mental state (i.e., cognitive and affective state) on DSS use. Line B: influence of DSSs on users' mental state (i.e., cognitive and affective state).

DSS efficiency could depend on both situational and environmental factors (Lee and Seong, 2009). We therefore assume that investigating the reciprocal influence of DSSs and operators' mental state (Figure 1) could provide useful avenues of investigation. Strong interest has been devoted to investigate technical, social, and cognitive factors influencing the adoption and use of systems (Stein et al., 2015). However, less interest has been devoted to the role of emotional factors in user behavior (Thüring and Mahlke, 2007; Stein et al., 2015). We will discuss some of the key findings in usability research aiming to understand how individuals and their mental state influence their engagement with information systems. We then aim to highlight the relative importance of investigating the influence of operators' emotional state on (re)scheduling decision-making. Particularly, we believe that the influence of unrelated emotions (i.e., incidental emotions) on feedback processing can account for the non-systematic improvement in operators' performance when using DSSs. Investigating the relative influence of DSSs and operators' mental state also involves characterizing the influence of DSSs on users' mental state. As a result, we will highlight how neuroergonomics can improve this latter line of investigation (Figure 1).

### Emotional Influences on DSS Use

Although research on usability is not new (e.g., Sagar and Saha, 2017 for a review), the interest to understand users' subjective experience while they interact with technological artifacts is more recent (e.g., Stein et al., 2015; Jung et al., 2017). It is then widely acknowledged that taking into account the feelings of users' experience is of crucial importance. Particularly, emotion could be one of the main dimensions of user experience (e.g., Thüring and Mahlke, 2007; Bargas-Avila and Hornbæk, 2011; Saariluomaand and Jokinen, 2014; Jeon, 2017). Studies looking at information technologies (e.g., DSSs) as an affect-inducing stimulus and influencing behavior (Figure 1, Line B) have to be distinguished from the ones looking at how affect influences information technology use (Figure 1, Line A; Stein et al., 2015). Subjective emotional experience shaped by the interaction with technologies depends on various factors related to the relationship between individuals' differences (e.g., coping, task events, and design) and emotions (Jokinen, 2015; Stein et al., 2015). A well-designed system is experienced as more positive, less arousing, more pleasant, goal conductive, and less novel (Thüring and Mahlke, 2007). According to Jokinen (2015), users appraise a significant task event and respond to it with coping strategies, which in return influence their performance (Jokinen, 2015).

Individuals' performance could also be affected by emotions induced prior to the interaction with information technologies. Although a large body of work investigates how information technologies induce emotions and how they influence subsequent behavior, little has been done in determining how incidental emotions (e.g., induced prior to the interaction) shape users' performance. A positive mood (or emotion) could be positively associated with a perceived ease of use (Cenfetelli, 2004), habit formation in technology use (Lankton et al., 2010), and appealing stimulus detection speed (Reppa et al., 2020). Aesthetic appeal could even counterbalance the effects of negative mood (Reppa et al., 2020). These results are in line with the fact that the emotional state under which individuals perform decision-making tasks modulates their performance (e.g., Blanchette and Richards, 2010). Users' affective state is largely ignored in the current DSS literature even if it has been demonstrated that individuals in a positive mood use their DSSs significantly more efficiently (Figure 1, Line A) than individuals in a neutral mood (Djamasbi, 2007). However, the authors take a valence-based approach (as the majority of usability research). This approach holds that all types of positive mood (or positive emotions) have the same influence, which differs from the performance triggered by all types of negative mood (or negative emotions; see Blanchette and Richards, 2010 for a review). The valence-based approach has been challenged by the seminal work of Lerner and Keltner (2000, 2001), who demonstrated that two emotions unrelated to the decision-making process, i.e., incidental emotions (e.g., induced prior to the decision-making tasks), and sharing the same valence could lead to different decisions.

This emotion-specific framework [i.e., The Appraisal Tendency Framework (ATF); Lerner and Keltner, 2001; see also Han et al., 2007] assumes that each incidental emotion can be defined by its score for a set of appraisals (pleasantness, anticipated effort, control, responsibility, attentional activity, and certainty, according to Smith and Ellsworth, 1985). For example, anger is defined by a high degree of certainty (i.e., the extent to which individuals understand what is happening and are able to predict future events; Smith and Ellsworth, 1985) and by individual control. By contrast, fear is defined by a low degree of certainty and situational control. These features could activate “a predisposition to appraise future events in line with the central appraisal dimensions that triggered the emotion” (Han et al., 2007, p. 160). A series of experiments showed that the appraisal of control associated with incidental emotions could mediate the link between emotions and decisions. Consequently, we posit that investigating the effect of incidental specific emotions could represent a fruitful avenue to deepen the understanding of operators' (re)scheduling decisions and the use of DSSs (Figure 1, Line A). It can therefore be expected that incidental-specific emotions influence the use of DSSs. For example, it can be hypothesized that incidental happiness leads to better DSS use than incidental hope, especially due to the different feedback processing triggered by those two incidental emotions (Mailliez et al., 2020).

### Emotional Influences and Feedback Processing

An explanation of why specific emotions could lead to different feedback processing—and thus performance—may stem from the type of information processing triggered by the appraisal of certainty (i.e., heuristic or deliberative processing). It has been shown that incidental negative emotions are associated with opposite patterns of performance (Bagneux et al., 2012, 2013; Bollon and Bagneux, 2013). Better performance is observed with emotions associated with a high degree of certainty (Bagneux et al., 2012, 2013; Bollon and Bagneux, 2013; Iyilikci and Amado, 2018). Particularly, incidental emotions associated with a high degree of certainty may trigger a heuristic feedback processing, whereas incidental emotions associated with a low degree of certainty might trigger a deliberative one (Tiedens and Linton, 2001).

When feedback is deliberatively processed, the number of times that a decision leads to a positive or negative outcome is used in controlled cognitive processes such as rethinking the decision strategy (Schiebener and Brand, 2015). Deliberative processing is strongly dependent on the individuals' available cognitive resources (Evans and Stanovich, 2013). Scheduling decisions are cognitively demanding and complex (Berglund and Karltun, 2007; Larsen and Pranzo, 2019). However, individuals' cognitive resources may be limited (Bechara and Damasio, 2005); hence, it could be impossible to define which decision is better than another. In contrast to deliberative processing, the heuristic processing of feedback might allow the processing of emotional cues shaped by the association between feedback (positive vs. negative outcomes) and the elicited emotions (Bechara and Damasio, 2005). Heuristic processing might enable the individuals to process emotionally charged information via the automaticity of emotions (Kahneman and Frederick, 2007). Heuristic processing is therefore less cognitively demanding and allows processing a greater amount of information. Consequently, the heuristic feedback processing might be more effective than deliberative processing to achieve better performance in sequential decision-making, such as (re-) scheduling ones.

While studies on feedback processing have largely demonstrated their influence on sequential decision-making (e.g., Brand et al., 2007; Schiebener and Brand, 2015), the effect of feedback processing on (re)scheduling has received little interest. Moreover, it has been stated that DSSs can replace or automate certain cognitive processes, leading to an increase in individuals' information processing capacities (Djamasbi, 2007). While DSS feedback studies highlight the effect of feedback on the use of DSSs (e.g., Lim et al., 2005; Djamasbi and Loiacono, 2008), they do not include the potential influence of specific emotions on feedback processing. Including such an incidental influence is particularly important as it can mediate the operators' performance. Particularly, emotional influences on feedback processing (Figure 1, interaction between Lines A and B) could be one of the factors explaining both better performance in (re)scheduling decisions and DSS efficiency, especially because emotional influences may trigger different information processing strategies (i.e., deliberative vs. heuristic).

To summarize, previous research on usability focused on how information technologies induce emotions and how these emotions influence the subsequent behavior (Figure 1, Line B). Influences of emotions that are not shaped by the interaction (i.e., incidental emotions) are less investigated (Figure 1, Line A), and interactions between these emotional influences even less so (Figure 1, interaction between Lines A and B). Scholars seem to focus on a specific period (e.g., during or before the interaction). It could be the consequence of a reluctance to theorize and operationalize the users' emotional experience (Jokinen, 2015). This reluctance might stem from the fact that user experience is considered as holistic (Boehner et al., 2007; Jokinen, 2015). This consideration and the valence-based approach taken by studies do not help to deepen the understanding of why and how users' emotional state and generally users' mental state influence the interaction with information technologies. We argued that taking an emotion-specific framework as forecasted by the ATF (and more recently, the emotion-imbued choice; Lerner et al., 2015) could provide an interesting path to consider both the contextual complexity (e.g., users' and technologies' characteristics) while taking into account the different emotional influences and their interaction. Beyond the theorization, operationalization could have everything to gain from being set in a neuroergonomic perspective.

### Toward Physiology-Based Mental State Assessment for DSS Use Characterization

Users' subjective experience can be thought of as private and immediate. The use of questionnaires to elicit users' emotional state has been widely debated (Schorr, 2001). One may argue that individuals are reporting their general knowledge concerning emotions, not their current emotional states (Jokinen, 2015). It could therefore be very difficult to put user experience into words (Dennett, 1988). Emotions (especially their associated appraisals) would be responsible for changes in individuals' physiology (Scherer, 2009). The investigation of emotional influences on (re)scheduling decisions can therefore take place within the larger context of deepening our understanding of DSS users' mental state through the lens of neuroergonomics.

Results about the effect of DSSs on users' mental state, especially their workload, remain equivocal, as it was mainly demonstrated at a subjective level. As Charles and Nixon (2019) highlighted in their systematic literature review, there is no single measure that discriminates mental workload, but there is a variety of physiological and behavioral data. A deeper understanding of the relationship between operators' mental workload using DSSs, as well as the reciprocal influence between DSSs and operators' mental state, will therefore be improved by the neuroergonomic approach. This approach is of particular interest as it allows going further than the classical approach based on subjective and behavioral measures by using physiological measures such as cardiac and cerebral activity markers. Indeed, behavioral metrics, although objective ones, might not reflect all mental processes that take place as illustrated by the inverted U-shaped performance curve observed under varying levels of arousal and task demands (VaezMousavi et al., 2009), as well as by the absence of difference reported between several difficulty levels for very low or very high task demands (Mehler et al., 2009). Hence, individuals may perform adequately but at a great cognitive cost, which might harm them in the long term, and impede their capability to deal with other task-external solicitations.

The neuroergonomic approach therefore allows assessing operators' mental state during operations (e.g., manufacturing ones) rather than afterward or by interrupting the task such as done with subjective measures acquired through questionnaires. Particularly, by using psychophysiological measures such as cardiac and cerebral activity ones, one could monitor operators' stress and workload level during (re)scheduling operations, as well as other cognitive and affective mental states as already studied in the ground and aerial transportation domains (see Borghini et al., 2014; Dehais and Callan, 2019 for reviews). Usual metrics for workload and stress assessment include heart rate and heart rate variability, as well as the power in various frequency bands (e.g., alpha power at parietal sites) recorded through electroencephalography (EEG; e.g., alpha [8 12] Hz) (Roy et al., 2013, 2020; Roy and Frey, 2016). Regarding affective state assessment, the same cardiac features (i.e., heart rate and heart rate variability), as well as the power in various EEG frequency bands and connectivity between electrodes (e.g., in the gamma band, >30 Hz), are metrics known to reflect arousal and valence (Wu et al., 2010; Chen et al., 2015). Usability studies that include physiological measurements have also started to be run (Hu et al., 2000; Brocke et al., 2013; Bhatt et al., 2019). It should be noted that physiological activity associated with mental processes can be recorded during operations (e.g., scheduling and rescheduling operations) and analyzed offline. However, the most striking advantage of this approach is that physiological measures can also be recorded and analyzed in an online manner.

Indeed, the use of such an online analysis approach has enabled researchers to deepen their mental states assessment in ecological settings, as well as to design better interfaces and support tools. Particularly, the use of machine learning tools has recently allowed researchers and engineers to develop adaptive systems that take physiological data as inputs. Such systems that enable cognitive and affective computing are often called biocybernetics systems or passive brain–computer interfaces (Fairclough, 2009; Zander and Kothe, 2011). By enabling the estimation of certain mental states (e.g., mental workload, fatigue, attentional level, emotional state) and modifying the interaction with the user, such bioadaptive or neuroadaptive systems provide a new means to increase safety and performance in operational environments (Lotte and Roy, 2019). Examples of countermeasures that could be implemented to deal with inadequate stress or mental workload levels are a modification of the interface, as performed in the Air Traffic Controller context (Aricò et al., 2016; Saint-Lot et al., 2020). Other solutions from the Human-Unmanned Aerial Systems interaction domain are to dynamically modify the automation level of the (re)scheduling task (Ruff et al., 2002) or even to dynamically reallocate the load between teammates (Walters and Barnes, 2002). To put in a nutshell, the neuroergonomic approach seems a fruitful avenue of investigation to deepen the understanding of the reciprocal influence between DSSs and users' mental state (Figure 1, Lines A and B).

## Conclusion

Although the complementary strength of DSSs and individuals has been demonstrated in previous work (see MacCarthy et al., 2001 for a review), to date there has been little work on characterizing the reciprocal influence of their mental state from an emotional perspective. Besides characterizing the influence of DSSs on users' mental state, factors such as the influence of incidental emotions and its interaction with feedback processing have received even less attention. However, they are of particular interest as they could represent factors that can improve individuals' and managers' performance. We argue that incidental emotions could mediate the effect of DSSs on users' mental state. This investigation cannot be carried out without considering the reciprocal influence of DSSs on mental (cognitive and emotional) state (and *vice versa*). As a perspective, the neuroergonomic approach is introduced. This approach is of particular interest for the human factors and the engineering communities that can benefit from new tools to better characterize (re)scheduling-induced mental states during DSS use. The striking advantage of this emerging approach is that it allows a psychophysiological assessment in both an offline and online manner.

## Data Availability Statement

The original contributions presented in the study are included in the article/Supplementary Material, further inquiries can be directed to the corresponding author.

## Author Contributions

MM contributed in the initial conception of the work, drafting the article, revisions of the article, and final approval of the version to be published. RR contributed in drafting the neuroergonomics sub-section, revisions of the article, and final approval of the version to be published. OB contributed in critical revisions of the article and final approval of the version to be published. All authors contributed to the article and approved the submitted version.

## Conflict of Interest

The authors declare that the research was conducted in the absence of any commercial or financial relationships that could be construed as a potential conflict of interest.
